# Prepubertal bipolar disorder: a diagnostic quandary?

**DOI:** 10.1186/s40345-020-00187-0

**Published:** 2020-04-20

**Authors:** Gin S. Malhi, Erica Bell

**Affiliations:** 1 Discipline of Psychiatry, Northern Clinical School, University of Sydney, Sydney, NSW Australia; 2 Department of Academic Psychiatry, Northern Sydney Local Health District, St Leonards, NSW Australia; 3 CADE Clinic, Royal North Shore Hospital, Northern Sydney Local Health District, St Leonards, NSW Australia; 4 CADE Clinic, Department of Psychiatry, Royal North Shore Hospital, Level 3, Main Hospital Building, St Leonards, NSW 2065 Australia

Duffy and colleagues (Duffy et al. 2020) (this issue) detail how differing perspectives that created controversy concerning the diagnosis of pre-pubertal bipolar disorder (PPBD), have in more recent times fostered a desire to find common ground. At the crux of this diagnostic dispute, lie a number of diagnostic requirements that build on the supposition that adult bipolar disorder begins early in life and therefore must manifest in childhood. To capture this earliest form that occurs prior to puberty, a couple of conditions need to be met. The first of these is that it should be a separate illness, and the second is that it’s trajectory should be distinguishable from normative development. Thus, the core assumption is that PPBD is a distinct illness in childhood with a unique profile that is maintained as it evolves into adult bipolar disorder.

However, the idea that PPBD is a separate and diagnosable disorder in childhood, is based on the observation that adult bipolar disorder (BD) is a distinct illness that is defined by episodes of mania and depression. Therefore, a similar episodic pattern should be evident early in the course of the illness and this can be used to define PPBD. But, the theoretical argument for this assumption is weak and empirical evidence is lacking. For example, it is clear that many of the symptoms of mania cannot be experienced by children in the same way as they are in adults, and therefore their expression is very different or lacking altogether. For example, heightened libido, grandiosity and diminished insight don’t have meaningful equivalents in children. Furthermore, many of the symptoms, thought to be distinctive of mania in adults, are less remarkable when they occur in children, and may even be regarded age-appropriate. For example, believing you are a superhero with special powers is of some concern in an 18-year-old but is perfectly normal in an 8-year-old. As a consequence, other symptoms have attracted interest, e.g. irritability. But even irritability, a seemingly familiar and all too common symptom, has proven to be a complex trans-diagnostic phenomenon that is difficult to characterise at any age (Toohey 2019; Toohey and DiGiuseppe 2017). Thus, the very first requirement that PPBD should be a diagnosable and distinct entity, poses a considerable challenge.

However, if for a moment we assume that PPBD is distinguishable in childhood, the subsequent requirement, namely that it pursues a trajectory separable from normative development, is equally problematic. Theoretically, such separation is conceivable, but if PPBD cannot be distinguished in childhood, the prospect of mapping its trajectory through puberty and into adolescence seems highly improbable.

A more fundamental difficulty that affects these considerations concerns whether BD in adults is indeed ‘bi-polar’. In other words, is bipolar disorder best characterised as an illness comprising two poles—mania and depression? Recently, the myriad clinical patterns of BD have been the focus of diagnostic debates (Malhi et al. 2018) and there has been a recrudescence of interest in mixed presentations, which are common in BD adults (Malhi et al. 2019). Hence, given the prevalence of adult mixed states, any childhood precursor of BD is also likely to have mixed symptoms (Malhi and Bell 2019). If this is the case, then it further complicates the transposition of clinical phenotype from adults to children and makes the prospect of identifying definable antecedents exceptionally challenging. This is especially so, given that mixed states are extremely poorly defined in adult bipolar presentations (Malhi 2013).

Hence, we remain at an impasse. And while we await a breakthrough that leads to greater knowledge, we suggest that greater efforts be made to conduct detailed longitudinal studies that carefully track phenomenology from childhood onwards through to adulthood without prior assumptions as to what bipolar disorder might look like in children.

## Data Availability

Not applicable
